# Performance of the Egoo test for phenylalanine measurement in females with phenylketonuria

**DOI:** 10.1186/s13023-025-03989-6

**Published:** 2025-10-10

**Authors:** Meriah S. Schoen, Serei Vatana Nath, Rani H. Singh

**Affiliations:** Department of Human Genetics, Emory University School of Medicine, Atlanta, GA USA

**Keywords:** Phenylketonuria (PKU), Phenylalanine (Phe), Home monitoring, Point-of-care-testing, Dried blood spots, Home blood sampling

## Abstract

**Background:**

Accurate measurement of phenylalanine (Phe) is a requirement for the diagnosis and management of individuals with Phenylketonuria (PKU). The current methods for quantifying Phe, plasma amino acids, and dried blood spots (DBS) have several analytical limitations and are not suitable for self-monitoring. This study evaluated the validity of a point-of-care testing (POCT) device, the Egoo Phe Test, in a sample of females with PKU who participated in a five-day camp intervention.

**Methods:**

Blood samples were collected from 20 participants (median age: 18 years; IQR: 16, 28.3) on the first (day 1) and/or final (day 5) day of camp for Phe quantification by plasma amino acids, DBS, and the Egoo Phe Test. Phe concentrations determined by these three methods were compared to assess agreement.

**Results:**

On both days of camp, strong positive agreement based on Lin’s concordance correlation coefficient (*p*_*c*_*)* was found between Egoo and DBS (day 1: *p*_*c*_ = 0.91 [95% CI*:* 0.78, 0.96]; day 5: *p*_*c*_ = 0.91 [95% CI: 0.79, 0.96]) and moderate positive agreement was identified between Egoo and plasma amino acids (day 1 *p*_*c*_ = 0.80 [95% CI: 0.63, 0.89]; day 5 *p*_*c*_ = 0.83 [95% CI: 0.68, 0.91]). Phe concentrations measured by the Egoo were lower than Phe concentrations determined by plasma amino acids (day 1 average difference: -31.2% [95% CI: − 40.2, − 25.8]; day 5 average difference: -12.4% [95% CI: -30.4, − 4.2]) and higher than DBS (day 1 average difference: 16.2% [95% CI: 4.6, 25.3]; day 5 average difference: 12.2% [95% CI: − 1.4, 20.5]). When assessing pairwise differences between the methods for each sample, > 66.7% of the Egoo Phe results were within ± 20% difference of the DBS values on day 5 of camp. However, this criterion was not met when comparing Egoo with DBS on day 1 of camp or with plasma amino acids on either day of camp.

**Conclusions:**

The Egoo Phe Test demonstrated potential as a substitute for DBS, but additional research in larger and more diverse clinical cohorts is required. Future research should focus on improving accuracy and defining analytical targets that consider the clinical impact of Phe test performance on management of patients with PKU.

**Clinical trial number:**

not applicable.

**Supplementary Information:**

The online version contains supplementary material available at 10.1186/s13023-025-03989-6.

## Background

Phenylketonuria (PKU, OMIM #261,600) is an inherited metabolic disorder (IMD) that causes reduced activity or complete deficiency of the hepatic enzyme phenylalanine hydroxylase, which metabolizes phenylalanine (Phe) to tyrosine [1]. The hallmark of PKU is elevated blood Phe concentrations, which can lead to neurocognitive sequelae when unmanaged [2]. To prevent these consequences, it is recommended that Phe levels are maintained within the therapeutic range of 120–360 µmol/L (2–6 mg/dL) through strict dietary Phe restriction, specialized medical foods (Phe-free or reduced-Phe amino acid-based formulas), and adjunct pharmacotherapies (e.g. Sapropterin Dihydrochloride, Pegvaliase) [3–5]. Frequent monitoring and dietary adjustment are critical for achieving good Phe control. This generally occurs twice weekly to monthly [4], depending on an individual’s age, disease severity, and treatment regimen.

Plasma amino acids collected via venous blood draws have traditionally been the gold standard for quantifying Phe concentrations [6]. However, this approach is typically utilized for diagnostic purposes or complete nutritional assessment due to venipuncture being more invasive and requiring a trained phlebotomist. The alternate approach, which involves spotting blood from heel sticks or finger pricks onto dried blood spot (DBS) cards is less invasive, requires less blood, and allows for home testing [7]. While these features have made DBS the standard of care for routine Phe monitoring, results can be affected by many factors. These include pre-analytical (cellulose/non-cellulose-based papers, specimen quality, drying and storage conditions), analytical (sample preparation, hematocrit concentrations, inter-laboratory variability), and post-analytical factors (large turnaround times) that can reduce the reliability and accuracy of the results [8–10]. Clinical application of these results can also be challenging given DBS Phe values are typically lower than plasma Phe values, with this bias increased in the lower Phe range [6, 11].

Beyond these factors, patients are dissatisfied with the current home sampling approach. This sentiment was found among 57% of patient and parent respondents (N = 352) in a recent survey from the United Kingdom, Netherlands, and Denmark, and led patients to collect samples less frequently than advised [12]. Much of this dissatisfaction stemmed from the slow turnaround time for results but could also reflect difficulties with the cost and efficiency of postal transport, which is required for DBS analysis. These challenges make timely treatment adjustments difficult for metabolic specialists [12]. Moreover, these obstacles have fueled a strong desire for point-of-care testing (POCT) devices, which could be used for efficient and accurate home monitoring. This opinion was confirmed by the same survey, which demonstrated that 97% of patient and parent respondents and 76% of professionals favored the use of POCT devices for Phe monitoring [12]. The utility of POCT devices also extends beyond home monitoring and may be used to improve the diagnostic odyssey, decrease the time to treatment for individuals with PKU and other IMDs, and enhance disease control, particularly in areas with limited resources.

To respond to this need, new chemical, optical, spectroscopic, and electrochemical methods have been developed to quantify Phe concentrations [13]. However, commercially viable POCT devices that leverage these analytical approaches have not yet been approved for clinical testing for PKU. The present study focuses on a new POCT device, the Egoo Phe Test (Egoo Health, Ballerup, Denmark). This device combines assay technology with a bioluminescent detection system to convert the light produced from a small blood sample into a Phe concentration. Phe results are then displayed on a smartphone app which is linked to the Egoo Device and allows users to easily access and track Phe levels. Given the sleek design, simple testing process, and rapid return of results (approximately 45 min), the Egoo Phe test offers great potential for enhancing the diagnosis and treatment of PKU. This study evaluated the validity of the Egoo Phe test compared to plasma amino acids and DBS in a sample of females with PKU who participated in a camp intervention with the objective of determining whether Egoo could serve as a viable alternative to DBS for routine home Phe monitoring for patients with PKU.

## Methods

### Sample and study design

Participants were recruited from the 2024 research-based Emory Metabolic Camp (Atlanta, GA), a five-day multifaceted experience for adolescent and adult females with PKU. This camp aims to improve disease management by providing group counseling, enrichment activities, educational seminars, and dietary supplies to support a Phe-restricted diet, including medical food and low-protein modified foods [14]. To evaluate the effectiveness of this intervention, changes in Phe concentration from the first (day 1) to the final day (day 5) of camp were evaluated for each camper. To be included in this study, camp participants needed to have a diagnosis of PKU, at least one Phe measurement, and provide written informed consent. All procedures were conducted in accordance with the ethical standards of the Emory University Institutional Review Board.

### Blood phenylalanine assessment

**Fig. 1:**
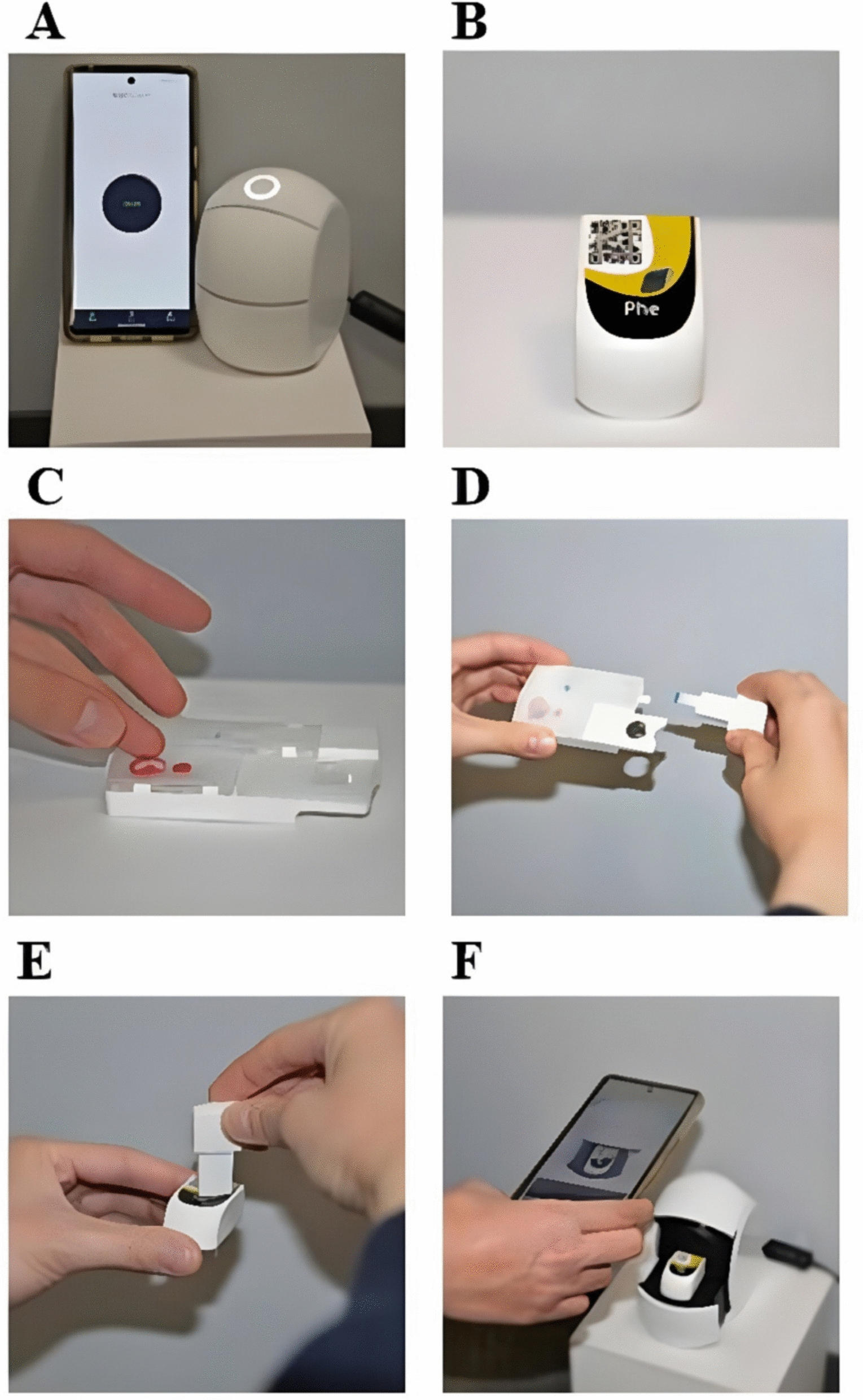
Procedure for the Egoo Phe test. **A** The Egoo Connect App is downloaded and installed on a mobile device, and on-screen instructions are followed to connect the mobile device to the Egoo Phe device (P300). Testing should proceed on a stable, flat surface. **B** An Egoo Phe capsule is removed from the freezer and defrosted for 10 min prior to testing. **C** Fingertip is punctured with a lancet and 2–3 full drops of blood are collected on the white membrane of the Egoo Collect board. **D** The Egoo Collect latch is closed, and plasma is filtered for 1–2 min. The test strip is removed when the white membrane turns green. **E** The test strip is inserted into the Egoo capsule. **F** The capsule containing the test strip is placed into the Egoo device and the mobile device is used to scan the QR code on the on the capsule. Analysis is started through the Egoo Connect app and takes approximately 30 min for the Phe result to be shown

To evaluate Phe concentrations on the first (day 1) and final (day 5) days of camp, fasting blood samples were collected at the Georgia Clinical and Translational Science Alliance Clinical Research Center at Emory University Hospital for adult participants and the Center for Advanced Pediatrics at Children’s Healthcare of Atlanta for pediatric participants. These samples were utilized for three blood testing methodologies: plasma amino acids, DBS, and the Egoo Phe Test. For plasma amino acids, 2 mL venous blood samples were collected in tubes containing sodium heparin as an anti-coagulant. After collection, blood samples were centrifuged at 1000 × g for 10 min to harvest plasma . Plasma samples were immediately frozen at − 80 °C and shipped to ARUP Laboratories (Salt Lake City, UT, USA) where Phe concentrations were determined using liquid chromatography-tandem mass spectrometry (LC–MS/MS). For DBS and the Egoo Phe Test, finger prick was performed using a lancet to collect whole blood. Fingers were massaged from their base and along their length to facilitate blood flow and ensure consistent sample quality. Two drops of blood were used to fill two DBS on a filter paper, which were dried completely and shipped to ARUP laboratories (Salt Lake City, UT, USA) for Phe determination via LC–MS/MS. Two drops of blood were additionally collected directly onto the Egoo Collect for the Egoo Phe test (Fig. 1). The Egoo Collect processes and filters the blood sample into a test capsule, which is inserted into the Egoo instrument. Each capsule consists of three reagent chambers and one cuvette that contains reagents, which are released by a piston in the Egoo instrument. Results take approximately 45 min to be displayed via the Egoo Connect smartphone app after completing the full collection and testing procedure.

### Principle of the Egoo Phe test

After the Egoo capsule is inserted into the Egoo instrument, the capsule, buffer and plasma sample are heated inside the Egoo device to 37 °C for 8 min to stabilize the temperature. Following this, the buffer, plasma and reagents are mixed and incubated for 20 min. During this phase, the Phe in the plasma is coupled to enzymatic Phe oxidation and NADH production is coupled to a bioluminescent NADH detection system (modified Glo-Assay, Promega, Madison, WI, USA). Phenylalanine dehydrogenase uses Phe and NAD + to produce NADH. In the presence of NADH, a pro-luciferin reductase substrate is converted by reductase to luciferin that is then used by a recombinant luciferase to produce light. The amount of light generated after a fixed time interval (20 min), or the luminescence end value ${L}_{End}$, is used to calculate Phe concentration in the blood sample using a pre-established calibration curve. The total Phe assay time is approximately 30 min.

### Calibration curve generation

Six concentrations of Phe were prepared in phosphate-buffered saline from a 1 mM Phe stock solution (Sigma- Aldrich P2126). Each of the 6 Phe concentrations was determined at ARUP Laboratories using LC–MS/MS (Table 1). Using 5 µL of each calibrator (CAL1-CAL5), the luminosity at the end of Egoo assay incubation or ${L}_{End}$ was determined as a function of the LC–MS/MS calibration values.

**Table 1:** Water calibrators used to determine luminosity at the end of Egoo assay incubation

Calibration solutions	Concentration of prepared Phe solution in PBS buffer (µmol/L)	Concentration of Phe solutions determined using LC–MS/MS (µmol/L)
CAL 1	0	0
CAL 2	50	58
CAL 3	100	114
CAL 4	500	466
CAL 5	1000	880
CAL 6	1500	1251

### ***Calculation of Phe concentration from luminescence value***${{\varvec{L}}}_{{\varvec{E}}{\varvec{n}}{\varvec{d}}}$

*End-of-Incubation Luminosity* (${L}_{End}$) refers to the raw light signal detected by the Egoo instrument at the end of the incubation period. This signal reflects the outcome of the biochemical reaction taking place in the test cartridge and correlates with the concentration of Phe in the sample. However, this raw signal may differ slightly between instruments due to inherent variations in photodiode sensitivity.

*Calibration Luminosity* (${L}_{Cal}$) is a predefined calibration constant stored in each Egoo device during manufacturing. It is determined by exposing the photodiode to a standardized light source and measuring its response. This value represents the baseline sensitivity of the light sensor in a given instrument and serves as a correction factor.

*Normalized Luminosity (L)* is calculated to correct for device-specific variability:

$$L=\frac{{L}_{End}}{{L}_{Cal}}$$

L = Luminosity.

${L}_{Cal}$ = Photo Diode (sensor) sensitivity in arbitrary units.

${L}_{End}$= The luminosity at the end of incubation.

This normalization allows the subsequent conversion of the signal to a phenylalanine concentration that is independent of the specific Egoo instrument used, ensuring reliable and reproducible results across clinical settings.

**Fig. 2:**
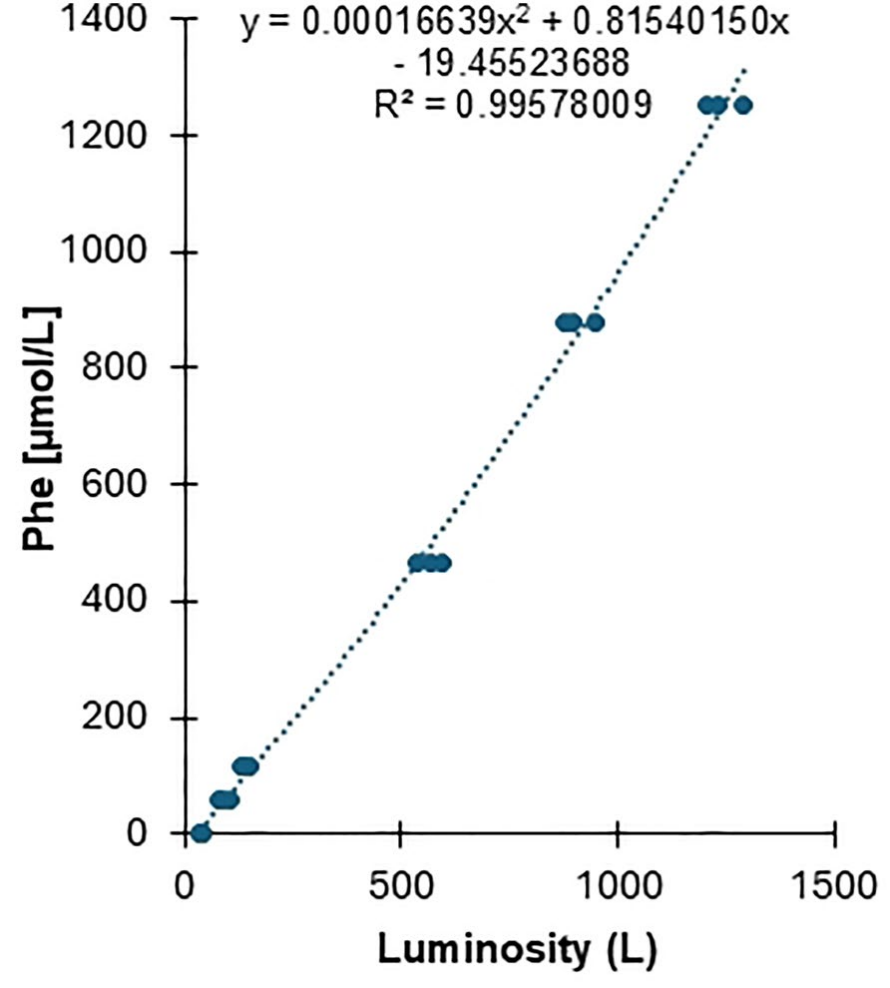
Second order polynomial calibration curve generated from six Phe calibrators. A luminescence value at the end of Egoo assay incubation (L_End_) was determined as a function of the LC–MS/MS values for each calibrator (Table 1). L_End_ was divided by a stored calibration value (L_cal_) to calculate a luminosity value (L) that is independent of the Egoo instrument

*Calculation of Phe Concentration* first involved model specification for the LC–MS/MS calibration values. The small sample corrected Akaike Information Criterion was utilized to specify the second order polynomial as the best-fit model for Phe calculation based on the LC–MS/MS calibration values. As illustrated in Fig. 2, the Phe calibrators were fitted to a second order polynomial so that Phe concentrations could be determined using the following formula:

$$Phe\left[ {\mu M} \right] = b \times L^{2} + c \times L + d$$

$$b \left( {\text {Coefficient of the quadratic term {L}}}^{2} \right) = 0.000{16639}$$

$$c \left( {\text{Coefficient of the linear term L}} \right) = 0.81540150$$

$$d \left( {\text{Constant term}} \right) = 19.45523688$$

$$L = {\text{luminosity}}$$

### Dietary assessment

Participants completed three-day dietary records in the three days preceding the first day of camp and for three days during camp which detailed all medical foods, foods, beverages, and supplements consumed. These records were reviewed for completeness and accuracy by trained registered dietitians and analyzed in MetabolicPro diet analysis software (Genetic Metabolic Dietitians International, Hillsborough, NC, USA).

### Statistical analysis

**Fig. 3:**
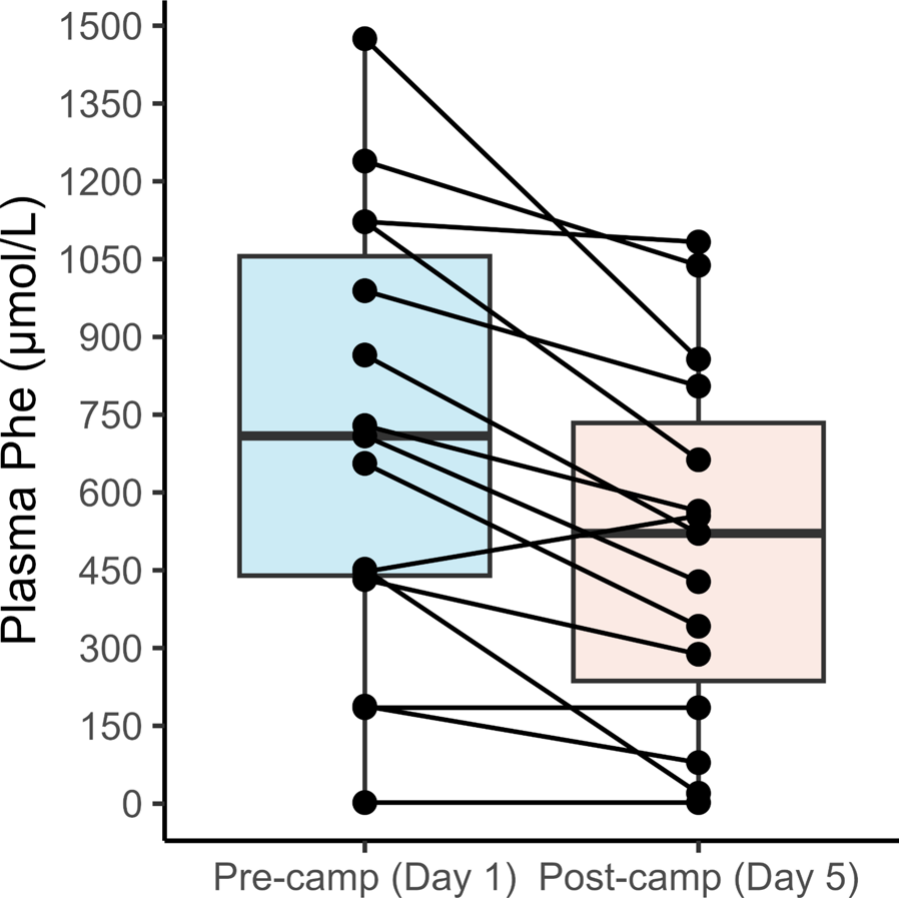
Changes in plasma Phe concentrations from pre-camp (day one) to post-camp (day five) in 15 females who had has plasma amino acids collected before and after the camp intervention. The Wilcoxon signed-rank test showed that there was a significant change in Phe concentrations following the camp intervention (Z = 89, p = 0.003)

Demographic, treatment, and dietary characteristics were reported as median (interquartile range [IQR]) for continuous variables and percentages for categorical variables. Using plasma amino acids as the gold standard approach, the Wilcoxon signed rank test was utilized to determine if there was a significant change in Phe concentrations with the camp intervention. Given this test demonstrated a notable decrease in Phe concentrations (Fig. 3), which is analogous to previous assessments of camp data [14], Phe values on the first and final days of camp were assessed independently for the remainder of study analyses.

The calculated Egoo Phe results for each camper were compared to their Phe values determined by plasma amino acids and DBS utilizing two main approaches: (1) Bland–Altman analysis, which determined the average bias and limits of agreement (LoA) [15, 16], and (2) Lin’s concordance correlation coefficient (*p*_*c*_), which quantified agreement [17]. For Bland–Altman Analysis, nonparametric LoA (2.5% and 97.5% percentiles) were reported given the distribution of differences did not meet normality criteria based on the Shapiro–Wilk test. To assess performance for clinical acceptability, a more stringent approach based on the International Conference on Harmonisation (ICH) M10 guidelines for incurred sample reanalysis (ISR) [18] was additionally utilized. Rather than using repeated measurements for the same sample, as proposed for ISR, the present study evaluated paired samples from the three Phe measurement methodologies. The following formula was employed to calculate differences between the Phe values from the Egoo Test relative to plasma amino acids and DBS:

$$\% \, difference=\frac{Egoo \,Phe -Reference \,Test \,Phe \,\left(Plasma \, Amino \,Acids\,or\, DBS\right)}{mean \,value } \times 100$$

Based on the acceptability criteria proposed for ISR, a difference of < 20% for at least 2/3 (66.7%) of the samples was utilized to determine if the Egoo Phe Test was a clinically acceptable substitute for plasma amino acids and DBS. R (version 4.4.2) was used for all statistical analyses, and P-values < 0.05 were considered statistically significant.

## Results

Baseline characteristics for the 20 female participants who met the eligibility criteria are reported in Table 2. Fifty percent of the females were adults and the median (IQR) age for the full sample was 18.0 years (16.0, 28.3). Most of the sample (55%) were managing their PKU with an adjunct pharmacotherapy, sapropterin dihydrochloride (35%) or pegvaliase (20%). The use of these therapies allowed many of the participants to exceed the typical intact protein consumption for individuals with classical PKU, which is usually less than 10 g per day [19]. At baseline (day 1 of camp), median total protein intake was 62.4 g/day (IQR: 47.5, 75.8), intact protein intake was 23.0 g/day (IQR: 16.9, 36.3) and Phe intake was 935.0 mg/day (IQR: 542.5, 1605.0). Based on plasma amino acids, 80% of the participants with Phe measures on both day 1 and day 5 (n = 15) experienced an improvement in metabolic control with the camp intervention. Consistent with previous years of camp, there was a significant decrease in Phe concentrations with a median (IQR) change of -184 µmol/L (-329, -74) (Z = 89, p = 0.003) (Fig. 3).

**Table 2:** Baseline demographic, dietary, and treatment characteristics of females attending the 2024 metabolic camp^1^

Age (Years)	18.0 (16.0, 28.3)
BMI (kg/m^2^)	27.4 (22.9, 33.7)
**Diet**	
Energy Intake (kcals/day)	1518.2 (1231.5, 1816.1)
Total Protein Intake (g/day)	62.4 (47.5, 75.8)
Intact Protein Intake (g/day)	23.0 (16.9, 36.3)
Phenylalanine Intake (g/day)	935.0 (542.5, 1605.0)
**Treatment**	
Diet Only	9 (45)
Sapropterin Dihydrochloride	7 (35)
Pegvaliase	4 (20)

^**1**^Values are median (IQR) or n (%)

### Comparison of blood Phe measurement methods

**Fig. 4:**
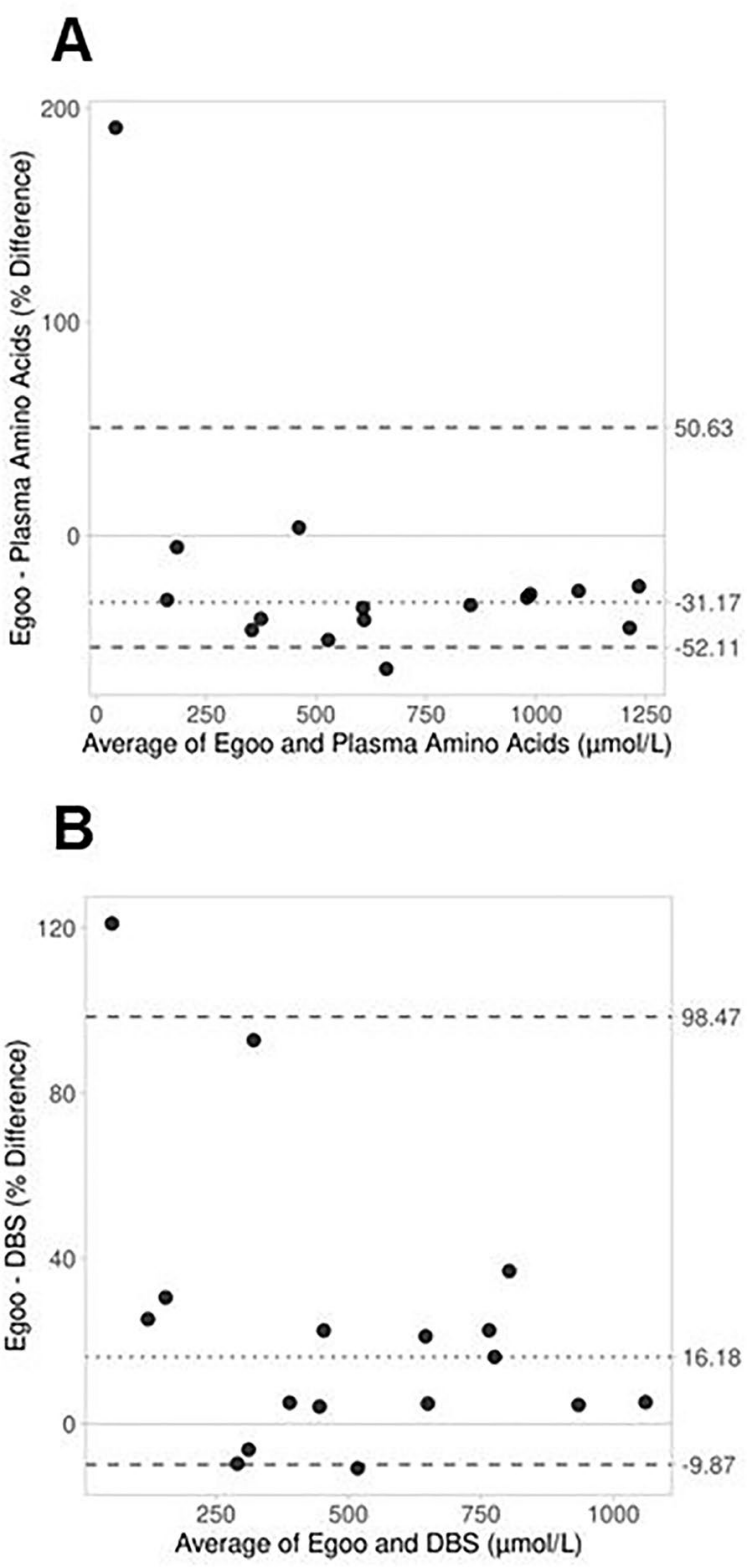
Bland–Altman plots comparing the Egoo Phe Test to plasma amino acids (A; n = 16) and DBS (B; n = 17) based on blood collected on day one of camp. The x-axis reflects the average concentration of Phe determined by the two methods and the y-axis represents the percent difference (difference/average *100) in Phe concentration between the methods (A: Egoo Phe Test-Plasma Amino Acids; B: Egoo Phe Test-DBS). The dotted line represents the average of each camper’s Egoo and plasma amino acid (**A**) or DBS (**B**) Phe measures. Large, dashed lines represent the upper and lower nonparametric limits of agreement

On day 1 of camp, 16 blood samples were collected for plasma amino acids and 17 were collected for DBS and the Egoo Phe Test. One participant refused venipuncture on both collection days of camp. Median (IQR) Phe concentrations were 719 µmol/L (443.3, 1122) based on plasma amino acids, 436 (304, 655) µmol/L based on DBS, and 489.4 (301.6, 838.6) µmol/L based on the Egoo Phe Test. Agreement between these methods was first evaluated using Bland Altman analysis, which revealed that Phe concentrations measured by Egoo Phe Test were on average 31.2% (95% CI: -40.2, -25.8) lower than the Phe measures determined by plasma amino acids, and two samples (12.5%) were outside of the LoA (-52.1, 50.6) (Fig. 4A). The bias between these methods was minimal when Phe concentrations were < 500 µmol/L but became more relevant as Phe concentrations increased. The opposite trend was observed when comparing DBS to the Egoo Phe Test. On average, Egoo Phe results were 16.2% (95% CI: 4.6, 25.3) higher than Phe concentrations measured via DBS (Fig. 4B), and this bias did not notably differ across the range of Phe concentrations. Among the 17 matched samples, two (11.8%) were outside of the LoA (-9.9, 98.5).

**Fig. 5:**
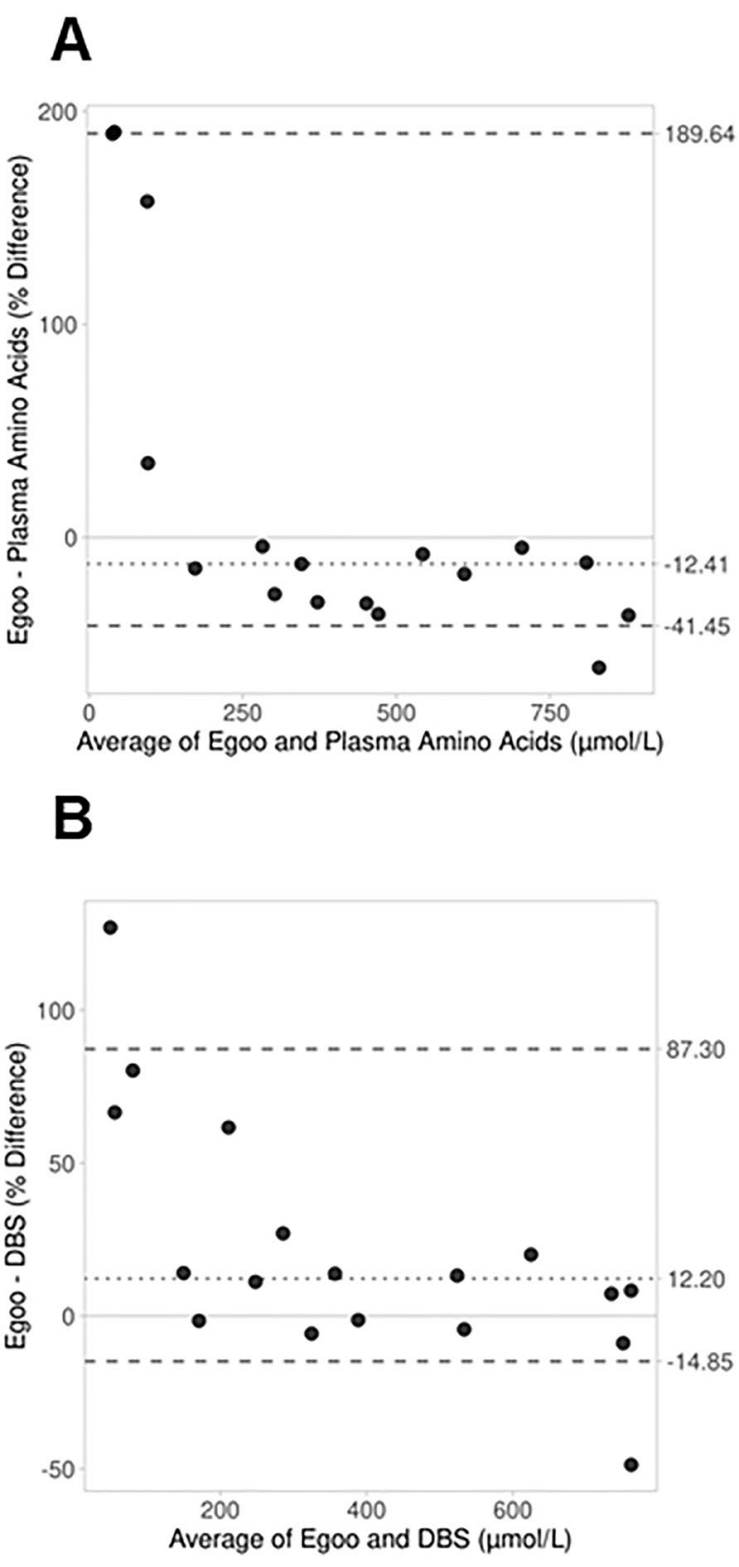
Bland–Altman plots comparing the Egoo Phe Test to plasma amino acids (A; n = 17) and DBS (B; n = 18) based on blood collected on day five of camp. The x-axis reflects the average concentration of Phe determined by the two methods and the y-axis represents the percent difference (difference/average *100) in Phe concentration between the methods (A: Egoo Phe Test-Plasma Amino Acids; B: Egoo Phe Test-DBS). The dotted line represents the average of each camper’s Egoo and plasma amino acid (**A**) or DBS (**B**) Phe measures. Large, dashed lines represent the upper and lower nonparametric limits of agreement.

Equivalent analyses were conducted for day 5 of camp. Eighteen blood samples were collected for plasma amino acids and 19 were collected for DBS and the Egoo Phe Test. One Egoo Phe result was discarded due to a leaky test capsule, leaving 17 paired samples for comparison with plasma amino acids and 18 paired samples for comparison with DBS. After the camp intervention, median (IQR) Phe concentrations were 428 µmol/L (185, 663) based on plasma amino acids, 333 µmol/L (152.5, 557.8) based on DBS, and 352.7 µmol/L (192.4, 571.8) based on the Egoo Phe Test. With the overall decrease in Phe concentrations by day 5 of camp, Bland Altman analysis showed a reduced bias for the Egoo Phe test relative to both methodologies. Phe concentrations measured by the Egoo were 12.4% (95% CI: -30.4, -4.2) lower than plasma amino acids and 12.2% (95% CI: -1.4, 20.5) higher than DBS, on average (Figs. 5A and B). Only one sample was outside the LoA (-41.5, 189.6) when comparing Egoo to plasma amino acids, and two samples were outside the LoA (-14.9, 87.3) when comparing Egoo to DBS.

The precision and accuracy of the Egoo Phe Test was further assessed using Lin’s Concordance Correlation Coefficient (*p*_*c*_; Table 3). There was positive agreement when comparing the Egoo Phe Test to both Phe measurement methods, but the concordance was stronger with DBS on both day 1 (*p*_*c*_ = 0.91 [95% CI*:* 0.78, 0.96]) and day 5 (*p*_*c*_ = 0.91 [95% CI: 0.79, 0.96]) of camp. Moderate agreement was found when comparing the Egoo to plasma amino acids (day 1 *p*_*c*_ = 0.80 [95% CI: 0.63, 0.89]; day 5 *p*_*c*_ = 0.83 [95% CI: 0.68, 0.91]).

**Table 3:** Correlations with Phe concentrations measured by the Egoo Phe Test^1^

	Camp Day 1		Camp Day 5	
	N	*p*_*c*_ (95% CI)	N	*p*_*c*_ (95% CI)
Plasma Amino Acids	16	0.80 (0.63, 0.89)	17	0.83 (0.68, 0.91)
DBS	17	0.91 (0.78, 0.96)	18	0.91 (0.79, 0.96)

^1^Correlation values are based on Lin’s Concordance Correlation Coefficient (*p*_*c*_*)*

Beyond assessing the range of agreement, the more stringent ICH criterion was applied to determine the clinical acceptability of the Egoo Phe results (Table 4). Compared to plasma amino acids, there was < 20% difference with Phe concentrations determined by Egoo for two samples (12.5%) on day 1 and seven samples (41.2%) on day 5. Most samples that were not acceptable (difference > 20%) on day 1 had Phe concentrations > 600 µmol/L, whereas the majority of failed samples on day 5 had Phe concentrations < 360 µmol/L. When applying this same criterion to compare Egoo results to DBS, Phe values for nine samples (52.9%) on day 1 and 12 samples (66.7%) on day 5 were considered acceptable. On both days, the majority of samples that were deemed not acceptable had Phe concentrations < 360 µmol/L. Across both methods (PAA and DBS), the majority of samples that met the ICH criteria had Phe concentrations 360–600 µmol/L.

**Table 4:** Acceptability of differences when comparing the Egoo Phe Test to plasma amino acids and DBS

	Camp Day 1		Camp Day 5	
	Plasma Amino Acids	DBS	Plasma Amino Acids	DBS
Number of Samples	16	17	17	18
Number Acceptable (%)^1^	2 (12.5)	9 (52.9)	7 (41.2)	12 (66.7)
Phe < 360 µmol/L (%)	1 (50.0)	1 (11.1)	2 (28.6)	5 (41.7)
Phe 360 – 600 µmol/L (%)	1 (50.0)	4 (44.4)	3 (42.9)	4 (33.3)
Phe > 600 µmol/L (%)	0	4 (44.4)	2 (28.6)	3 (25.0)
Number Not Acceptable (%)	14 (87.5)	8 (47.1)	10 (58.8)	6 (33.3)
Phe < 360 µmol/L (%)	2 (14.3)	4 (50.0)	5 (50.0)	5 (83.0)
Phe 360 – 600 µmol/L (%)	2 (14.3)	2 (25.0)	3 (30.0)	0
Phe > 600 µmol/L (%)	10 (71.4)	2 (25.0)	2 (20.0)	1 (16.7)

^1^A difference in paired Phe measurements < 20% was deemed acceptable

## Discussion

It is well-recognized that adherence to recommendations for blood testing frequency and blood Phe concentrations is suboptimal among individuals with PKU, and particularly among older patients [20]. Prior investigations have found that 62–68% of patients younger than 18 years of age and 23–26% of adults had Phe concentrations in the recommended 120–360 µmol/L range [20, 21]. While blood Phe control has improved over time with the establishment of stricter guidelines [22], increased variety of low-protein modified foods [23], and the introduction of adjunct pharmacotherapies [24, 25], there is still a pressing need for innovative approaches that can facilitate better disease management. POCT devices, such as the Egoo Phe Test, may immensely improve diagnosis and monitoring for individuals with PKU. With simple collection procedures and the rapid determination of Phe concentrations, POCT devices have the potential to revolutionize newborn screening by removing the need for specialized laboratory facilities and personnel. This could fill an essential gap in low resource areas and allow for quicker initiation of treatment. POCT devices also have the potential to reduce or eliminate critical barriers associated with current standard-of-care approaches for lifelong Phe measurement, including pre-analytical variability [8], long turnaround time for results [9], and restricted self-monitoring [26]. By removing these challenges, it is expected that individuals with PKU will more frequently measure blood Phe using POCT devices [12], which will enhance self-management [27] and ultimately metabolic control [28].

While POCT devices represent a new landscape for PKU, they have already proven to be beneficial for managing other IMDs, such as glycogen storage disease (GSD) [29]. For both children and adults with GSDs, the use of continuous glucose monitors (CGMs) has improved glycemic control by identifying valuable trends in hypo- and hyperglycemia that could previously only be detected in inpatient settings [30, 31]. These findings have aided providers in making timelier and more effective dietary modifications, while also helping patients better understand their condition, take greater initiative in their disease management, and improving their quality of life [30]. Given POCT devices for PKU will have the unique capability of capturing trends, like CGMs for GSDs, they can provide valuable insight on Phe fluctuations. A better understanding of these patterns is critical for optimizing cognitive outcomes given previous studies have demonstrated that minimizing Phe fluctuations is just as important as maintaining low blood Phe levels [32, 33]. This additional information will aid clinical providers in further personalizing medical and nutritional management for PKU.

The present study evaluated the performance of the Egoo Phe Test, an innovative POCT device, in a sample of adolescent and adult females with PKU who were participating in a five-day camp. The Phe values determined by the Egoo were compared to the standard methodologies of Phe measurement, plasma amino acids and DBS, both before (day 1) and after (day 5) the camp intervention. During the collection days of camp, Phe concentrations determined by the Egoo were generally lower than the Phe values measured by plasma amino acids and higher than the results from DBS. Considering both days of camp, the average bias ranged from -31.2% to -12.4% when comparing Egoo Phe results to plasma amino acids, and 16.2% to 12.2% when comparing Egoo to DBS. While these differences may exceed 10.4%, which is the acceptable bias based on the biological variation of Phe [34, 35], they are less than the differences between plasma amino acids and DBS found in the present study. Even when using the same analytical method (LC–MS/MS) for quantification, plasma amino acids were, on average, 46.9% (95% CI: 32.8, 54.4) higher than DBS on day one of camp and 27.9% (95% CI: 13.4, 37.5) higher than DBS on day five of camp (Additional File 1, Fig. A1). This trend aligns with many previous studies, which have found that DBS is generally 15–28% lower than plasma amino acids [9]. Given the average differences that we found between Egoo and DBS did not exceed this range, the Egoo Phe Test may be an appropriate substitution for DBS. This is further supported by our concordance correlation analysis, which found strong positive agreement between the methodologies.

There is presently no optimal analytical target for Phe testing that considers the impact of test performance on clinical outcomes among individuals with PKU [34]. To gauge clinical acceptability in the present sample, the ICH guidelines for ISR were applied. ISR is typically employed in clinical trials to validate an analytical method and involves retesting a subset of samples to verify the reliability, quality, and consistency of the measurements. Using the same criterion (percent difference within 20% for at least 66.7% of samples) to compare the Egoo Phe measures to those determined by plasma amino acids and DBS, we found that the Egoo achieved the ISR target when compared to DBS on day five of camp. Most samples that significantly differed (> 20%) between Egoo and DBS on both days of camp had a Phe concentration < 360 µmol/L, suggesting that the Egoo Phe test may not be as sensitive as DBS for capturing Phe levels in the normal range or in the lower end of the recommended therapeutic range. This finding was consistent when comparing Egoo Phe results to plasma amino acids, in that 50% of the samples on day 5 of camp which failed to meet the ISR criterion had Phe concentrations < 360 µmol/L. Although the Egoo Phe results did not meet the ISR target when compared to plasma amino acids, the percentage of acceptable samples (difference < 20%) on both days was higher than found in a supplemental analysis of DBS compared to plasma amino acids (Additional File 1, Table A1). For this comparison, acceptability criterion was achieved by no samples on day 1 of camp and only 17.6% of samples on day 5 of camp.

While these analyses provide preliminary evidence to suggest that the Egoo Phe Test can be utilized in place of DBS for Phe measurement, this study has some important limitations that presently limit clinical translation. First, generalization of study results to the PKU population is limited by our relatively small sample that solely included females. While future studies will benefit from larger samples with greater phenotypic variability, the present study was strengthened by data collection at two time points and the inclusion of four participants managed with pegvaliase. Two of these participants had plasma Phe levels that were < 30 µmol/L and the Egoo was not able to accurately measure these concentrations (> 20% difference), per the ISR criterion. However, the low plasma Phe levels may reflect residual pegvaliase activity in the blood specimens rather than actual hypophenylalaninemia [36], which make the plasma results potentially unreliable as a reference among patients on this treatment. Nevertheless, the Egoo did underperform in other campers with blood Phe concentrations < 360 µmol/L who were not treated with pegvaliase. This may be attributed to the original calibration approach used for the Egoo, which was based on buffer-derived Phe standards fitted to a second-order polynomial model. While this approach was appropriate for early development, it may not fully account for biological matrix effects at lower Phe levels, leading to the observed underperformance in that range. As this could adversely affect patient management, particularly among women with PKU who are pregnant, Egoo Health is actively working to expand its dataset by incorporating more patient samples at lower concentrations, which will support the development of matrix-matched calibration models. These future models will employ more advanced curve-fitting strategies, such as segmented or spline regression, which allow for greater flexibility and accuracy across the full clinical range. Another limitation is that this study did not systematically gather data on the useability and practicality of the Egoo device from the participant perspective given the test was administered by trained researchers in a camp environment. Despite the lack of formal assessment, most campers shared positive feedback about the testing experience and felt that it would be easy to use the device regularly at home. One camper who was pregnant further expressed how the Egoo would significantly reduce the anxiety she experiences while waiting for DBS or plasma amino acid results. Future studies will benefit from incorporating a structured useability assessment to more comprehensively examine the patient experience while using the Egoo Phe Test and other POCT devices.

## Conclusions

This study provides foundational evidence on the reliability and accuracy of the Egoo Phe test relative to plasma amino acids and DBS. Our findings suggest that the Egoo Phe test is a viable alternative to DBS for home monitoring of phenylalanine in individuals with PKU. However additional research in larger and more diverse clinical cohorts that include males and young children is required to refine accuracy and assess long term clinical utility. Standardized analytical targets that consider the impact of test performance on clinical outcomes in PKU are needed to optimize future testing.

## Supplementary Information

Additional file1

## Data Availability

The datasets used and/or analyzed during the current study are available from the corresponding author on reasonable request.

## Abbreviations

DBS: Dried blood spot
ICH: International conference on harmonisation
IMD: Inherited metabolic disorder
ISR: Incurred sample reanalysis
LC–MS/MS: Liquid chromatography-tandem mass spectrometry
LoA: Limits of agreement
PKU: Phenylketonuria
Phe: Phenylalanine
POCT: Point-of-care testing
