# Supplementary material for: Performance of the Egoo test for phenylalanine measurement in females with phenylketonuria
Source: Orphanet J Rare Dis. 2025 Oct 10;20:510. doi: 10.1186/s13023-025-03989-6 (PMC12512701; doi:10.1186/s13023-025-03989-6)
Supplement: Supplementary file 1 — Additional file1 [file 13023_2025_3989_MOESM1_ESM.docx]

**Additional File 1**

**
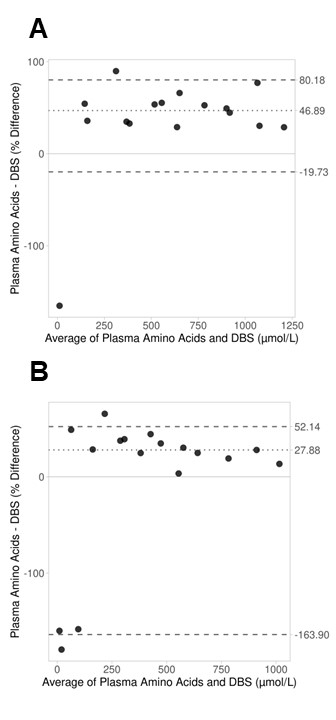
**

**Figure A1.** Bland-Altman plots comparing plasma amino acids to DBS based on blood collected on day one (A; n=16) and day five (B; n=17) of camp. The x-axis reflects the average concentration of Phe determined by the two methods and the y-axis represents the percent difference (difference/average *100) in Phe concentration between plasma amino acids and DBS. The dotted line represents the average of each camper’s Phe results from the two methods. Large, dashed lines represent the nonparametric upper and lower limits of agreement.

**Table A1. Acceptability of differences when comparing Phe concentrations measured by DBS to plasma amino acids.**

|  | Camp Day 1 | Camp Day 5 |
| --- | --- | --- |
| Number of Samples | 16 | 17 |
| Number Acceptable (%)^1^ | 0 (0) | 3 (17.6) |
| Phe < 360 µmol/L (%) | 0 | 0 |
| Phe 360 – 600 µmol/L (%) | 0 | 1 (33.3) |
| Phe > 600 µmol/L (%) | 0 | 2 (66.7) |
| Number Not Acceptable (%) | 16 (100) | 14 (82.4) |
| Phe < 360 µmol/L (%) | 3 (18.8) | 7 (50.0) |
| Phe 360 – 600 µmol/L (%) | 3 (18.8) | 3 (21.4) |
| Phe > 600 µmol/L (%) | 10 (62.5) | 2 (14.3) |

^1^A difference in paired Phe measurements <20% was deemed acceptable.
